# Argyrophilic Nucleolar Organizer Regions as New Biomarkers in ST-Elevation Myocardial Infarction

**DOI:** 10.3390/jcdd9020058

**Published:** 2022-02-14

**Authors:** İbrahim Halil Damar, Recep Eroz

**Affiliations:** 1 Department of Cardiology, Medical Faculty, Duzce University, Duzce 81820, Turkey; 2 Department of Medical Genetics, Medical Faculty, Aksaray University, Aksaray 68100, Turkey; receperoz@aksaray.edu.tr

**Keywords:** AgNOR, hypoxia, ischemia, STEMI

## Abstract

(1) Background: ST-elevation myocardial infarction (STEMI) is an inflammatory disease in which neutrophils, macrophages, and lymphocytes accumulate in the ischemic myocardium and have important functions. Nucleolar-organizing regions (NORs) are the site of the ribosomal genes composed of ribosomal DNA and proteins. We aimed to evaluate AgNOR proteins, which have never been studied in patients with STEMI in the literature. (2) Methods: A total of 140 participants (75 with STEMI and 65 volunteers without any diagnosis of acute coronary syndrome) were included in this study. Echocardiography was carried out, and mean AgNOR number and total AgNOR area/total nuclear area (TAA/TNA) were evaluated for all individuals. (3) Results: The mean AgNOR number and TAA/TNA ratio were significantly higher in the STEMI group than the control (*p* < 0.001). Statistically significant relations between both TAA/TNA ratio and mean AgNOR number and interventricular septal thickness, fasting blood sugar, creatinine, HDL, hemoglobin (g/dL), WBC (µL/mL), monocytes, neutrophils, and neutrophil/lymphocyte ratio were detected (*p* < 0.05). Moreover, a statistically significant relation between LDL (mg/dL) and mean AgNOR number (*p* = 0.005) was detected. (4) Conclusion: Both AgNOR protein amounts increase depending on the hypoxia that occurs in STEMI. The AgNOR proteins may thus be promising markers in STEMI.

## 1. Introduction

Acute myocardial infarction is an inflammatory disease that usually develops on the basis of coronary artery disease [1]. Immune system cells, which infiltrate into the plaque from the early stages of atherosclerosis, increase inflammation by synthesizing and stimulating the molecules that accelerate the progression of the lesion [2]. During the acute myocardial infarction process, within the first five hours after reperfusion, neutrophils, as well as macrophages and lymphocytes, accumulate in the ischemic myocardium [3]. T lymphocytes have a key role in the pathophysiology of acute myocardial infarction (MI) within the cells of the immune system. T cells activate B lymphocytes, monocytes, macrophages, endothelial cells, and smooth muscle cells by releasing many cytokines, such as interferon-gamma [4,5].

Nucleolar-organizing regions (NORs) are the site of the ribosomal genes composed of ribosomal DNA (rDNA) and proteins; some of them have argyrophilic features. After silver staining, NORs can especially be localized as black spots along the nucleolar space and are termed “AgNOR”. Different studies on the importance of AgNOR proteins were performed in various cells such as hair root cells [6], buccal epithelial cells [7], lung cells [8], myocytes [9,10], muscle cells [11], etc. In those studies, the levels of AgNOR protein increased depending on the hypoxic condition caused by CO exposure and may have had protective effects against hypoxic conditions [9,10].

In MI, myocardial ischemia/hypoxia occurs depending on acute coronary thrombosis. To the best of our knowledge, there are no studies evaluating AgNOR proteins in patients with MI. Thus, in the present study, we aimed to compare the AgNOR values of patients with ST-elevation myocardial infarction (STEMI) with those of people without acute coronary syndrome.

## 2. Materials and Methods

### 2.1. Study Design

A total of 140 participants who presented to the outpatient cardiology clinic of our institution were included in the study. The STEMI group consisted of 75 patients with a diagnosis of STEMI who underwent percutaneous coronary intervention, and the control group consisted of 65 volunteers without any diagnosis of acute coronary syndrome. The study protocol was approved by the local ethics committee (ethical approval code: 2021/62). Written informed consent was obtained from the participants. Uncontrolled hypertension, congenital heart disease, atrial fibrillation, severe valvular heart disease, hypothyroidism, hyperthyroidism, malignancy, and infection were determined as exclusion criteria. The diagnosis of STEMI was made under the guidance of the Fourth Universal Definition of Myocardial Infarction [12]. In patients who underwent percutaneous coronary intervention, primarily, the procedure only intervened in the responsible total lesion. Coronary artery stenosis was determined if the plaques caused 50% or more obstruction in the coronary lumen, while hemodynamically insignificant stenosis was determined if the lesions caused less than 50% stenosis. Blood was obtained from patients included in the study with a diagnosis of STEMI within the first six hours after the onset of chest pain. Diabetes mellitus was defined by use of antidiabetic therapy or fasting plasma glucose levels of >6.94 mmol/L (>125 mg/dL). Hypertension was defined as antihypertensive drug use or blood pressure of ≥140/90 mmHg. Hyperlipidemia was defined as serum low-density lipoprotein level of ≥2.6 mmol/L, triglycerides of ≥1.7 mmol/L, total cholesterol of ≥5.2 mmol/L, or the use of cholesterol-lowering drugs (1). Smokers were defined as people who continue to smoke currently. Laboratory findings (creatinine, low-density lipoprotein (LDL), high-density lipoprotein (HDL), triglyceride, total cholesterol level, and complete blood cell count (CBC)) and demographic features of the participants were recorded.

### 2.2. Electrocardiography and Echocardiography

Resting 12-lead ECGs of all patients were recorded using the NIHON KOHDEN Cardiofax ECG 1250K model (filter range, 0.05–150 Hz; AC filter, 60 Hz, 25 mm/s 10 mm/mv). Echocardiography of the patients was performed using a Siemens Acuson SC2000 device. Cardiac anatomy, segmental wall motion abnormality, ejection fraction, and valve function were evaluated in accordance with the recommendations of the American Echocardiography Association with standardized projections [13].

### 2.3. Coronary Angiography

Selective right and left coronary angiography and PCI procedures were performed on patients in the STEMI group using the standard Judkin technique with a General Electric INNOVA 2100 IQ device. Coronary arteries were visualized in right and left oblique positions with cranial and caudal angulations.

### 2.4. AgNOR Staining

Blood samples of both the control and STEMI groups were taken and spread on clean slides. The slides were air dried for 15 min and fixed in absolute methanol for 5 min at room temperature. After that, the silver staining method with slight modification of the Benn and Perle protocol [14] and Lindner’s technique [15] was performed for each slide. The solution, made by mixing one volume of 2% gelatin in 1% aqueous formic acid and two volumes of 50% silver nitrate, was dropped on the slides and incubated at 37 °C for 15 min in the dark. After the incubation, the slides were rinsed with double distilled water.

### 2.5. Image Analysis of Mean AgNOR Number and Total AgNOR Area/Total Nuclear Area (TAA/TNA) Ratio

Fifty nuclei for each slide were evaluated. Firstly, silver-stained lymphocyte cells of each individual were photographed using a light microscope (Eclipse 80i; Nikon, Tokyo, Japan) with an attached digital camera (Digital Sight DS-Fi1c; Nikon). Then, each nucleus was evaluated using ImageJ version 1.47t image processing software [16] to determine both the TAA/TNA ratio and the mean AgNOR number via the “freehand selection” tool for each nucleus.

### 2.6. Statistical Analysis

The research data were uploaded and analyzed using the Statistical Package for Social Sciences (IBM Corp., Armonk, NY, USA) version 23.0. The distribution of the data was examined using the Kolmogorov–Smirnov test. The independent samples *t*-test and Mann–Whitney U test were used in comparisons of the variables with and without normal distribution, respectively. The descriptive statistic and Mann–Whitney U tests were used for pairwise comparison of groups. Additionally, a polynomial regression test was performed. Moreover, Bayesian statistics based on the receiver operating characteristic (ROC)-derived cut-off values were calculated. *p* < 0.05 was accepted as statistically significant.

## 3. Results

In total, 140 individuals (75 with STEMI and 65 as the control) were included in the current study. The male and female sex frequency was 80% and 20% for both groups (*p* = 1). Among the laboratory findings of the groups, the fasting blood sugar, creatinine (mg/dL), WBCs (white blood cells) (µL/mL), monocytes, neutrophils, and neutrophil/lymphocyte ratio were significantly higher in the STEMI group than in the control group (*p* < 0.05 for all) (Table 1). Conversely, LDL (mg/dL), HDL (mg/dL), and hemoglobin (g/dL) were significantly lower in STEMI patients than in the control group (*p* < 0.05 for all) (Table 1). According to the echocardiographic findings, while the interventricular septum thickness (IVST) was significantly higher in the STEMI group (*p* < 0.001), conversely, the ejection fraction (EF) value was significantly lower in the STEMI group (*p* < 0.001). Moreover, both mitral regurgitation (MR) and tricuspid regurgitation (TR) were significantly higher in the STEMI group than in the control (*p* < 0.05 for all) (Table 1).

**Figure 1:**
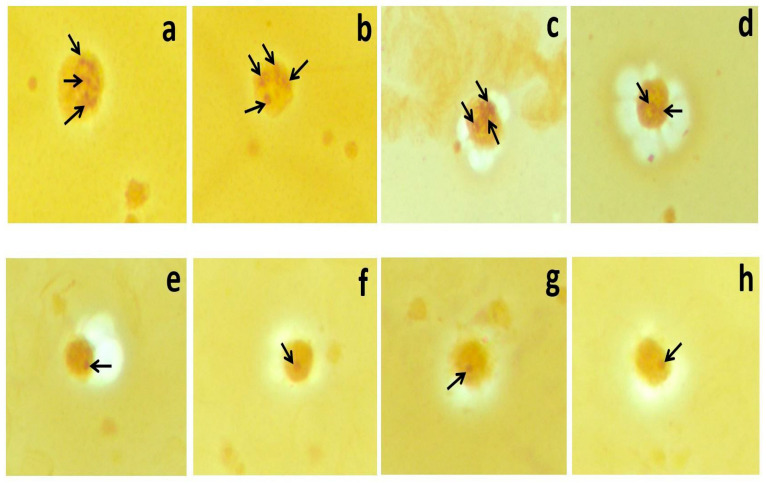
Silver-stained NORs in the lymphocytes of STEMI (**a**–**d**) and control (**e**–**h**) group members (×100 magnification).

Moreover, the mean AgNOR number (2.56 ± 0.8 vs. 1.32 ± 0.49) and TAA/TNA ratio (0.11 ± 0.03 vs. 0.03 ± 0.01) were significantly higher in the STEMI group than in the control (*p* < 0.001). Silver-stained NORs in the lymphocytes of STEMI (a, b, c, and d), and control (e, f, g, and h) group members (×100 magnification) are shown in Figure 1.

**Figure 2:**
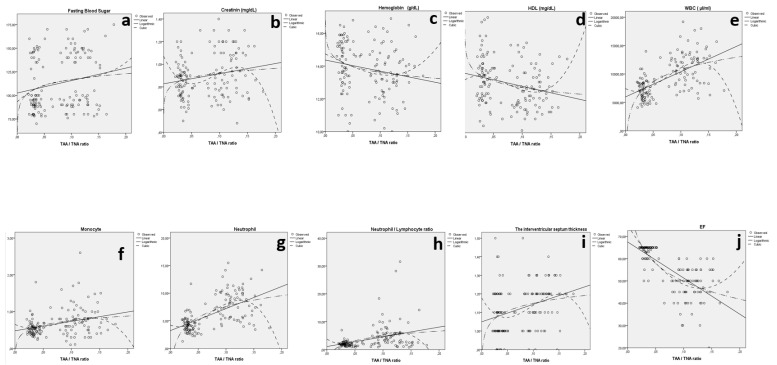
The relation between fasting blood sugar and TAA/TNA ratio (**a**), creatinine and TAA/TNA ratio (**b**), hemoglobin and TAA/TNA ratio (**c**), HDL and TAA/TNA ratio (**d**), WBCs and TAA/TNA ratio (**e**), monocytes and TAA/TNA ratio (**f**), neutrophils and TAA/TNA ratio (**g**), neutrophil/lymphocyte ratio and TAA/TNA ratio (**h**), IVST and TAA/TNA ratio (**i**), and EF and TAA/TNA ratio (**j**) for both groups.

When the TAA/TNA ratio was considered, statistically significant relations between IVST and TAA/TNA (*p* < 0.001), fasting blood sugar and TAA/TNA (*p* = 0.020), creatinine and TAA/TNA (*p* = 0.014), HDL (mg/dL) and TAA/TNA (*p* = 0.002), hemoglobin (g/dL) and TAA/TNA (*p* = 0.012), WBCs (µL/mL) and TAA/TNA (*p* < 0.001), monocytes (×10^3^) and TAA/TNA (*p* < 0.001), neutrophils (×10^3^) and TAA/TNA (*p* < 0.001), neutrophil/lymphocyte ratio and TAA/TNA (*p* < 0.001), and EF and TAA/TNA (*p* < 0.001) were detected (Figure 2 and Table 2).

**Figure 3:**
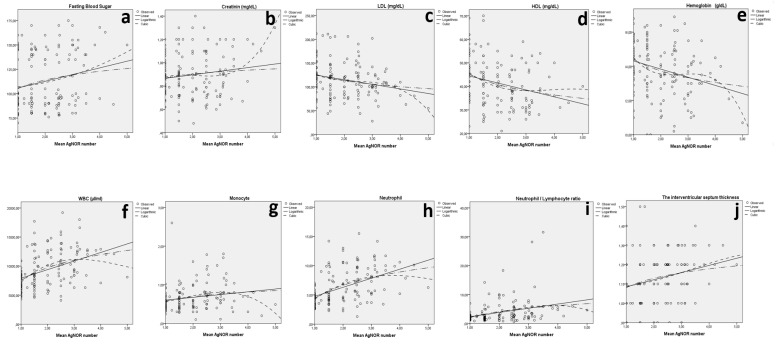
The relation between fasting blood sugar and the mean AgNOR number (**a**), creatinine and the mean AgNOR number (**b**), LDL and the mean AgNOR number (**c**), HDL and the mean AgNOR number (**d**), hemoglobin and the mean AgNOR number (**e**), WBCs and the mean AgNOR number (**f**), monocytes and the mean AgNOR number (**g**), neutrophils and the mean AgNOR number (**h**), neutrophil/lymphocyte ratio and the mean AgNOR number (**i**), and IVST and the mean AgNOR number (**j**) for both groups.

Additionally, when the mean AgNOR number was considered, statistically significant relations between IVST and the mean AgNOR number (*p* = 0.002), fasting blood sugar and the mean AgNOR number (*p* = 0.011), creatinine and the mean AgNOR number (*p* = 0.033), LDL and the mean AgNOR number (*p* = 0.005), HDL and the mean AgNOR number (*p* < 0.001), hemoglobin (g/dL) and the mean AgNOR number (*p* = 0.001), WBC (µL/mL) and the mean AgNOR number (*p* < 0.001), monocytes and the mean AgNOR number (*p* = 0.010), neutrophils and the mean AgNOR number (*p* < 0.001), neutrophil/lymphocyte ratio and the mean AgNOR number (*p* < 0.001), and EF and the mean AgNOR number (*p* < 0.001) were detected (Figure 3 and Table 3).

The troponin values of patients were 40.4 ± 22.3. Additionally, when both the mean AgNOR number and the TAA/TNA ratio were considered, there were no statistically significant relations between the levels of troponin and both AgNOR amounts (mean AgNOR number and TAA/TNA) (*p* > 0.05). The Bayesian statistics based on the receiver operating characteristic (ROC)-derived cut-off values are shown in Table 4.

## 4. Discussion

The present study showed that both the TAA/TNA ratio and the mean AgNOR number were significantly higher in the STEMI patients compared to the subjects without acute coronary syndrome.

In our previous studies, we detected that the AgNOR proteins increased depending on the hypoxic condition caused by CO exposure [9]. At the same time, it was reported that the TAA/TNA ratio could be used instead of histopathological evaluation scores as a biomarker for obtaining knowledge about the myocardial damage degree in rats [9]. Moreover, we reported that the TAA/TNA ratio could be used as an indicator for detection of the CO exposure level that causes hypoxia [17]. Additionally, we learned that knowledge about cardiomyopathy (CM) levels may be obtained via AgNOR proteins, and these proteins may be used instead of carboxyhemoglobin (COHb) to detect CO intoxication levels [10]. In acute coronary thrombosis, myocardial ischemia/infarction, and the reperfusion process, it is highly probable that the molecular protective mechanisms of immune system cells will be activated. In our study, because the mean AgNOR number and TAA/TNA ratios were significantly higher in STEMI patients compared to the control group, it may be considered a protective reaction of the immune system in the early period of MI.

When the TAA/TNA ratio was taken into consideration, statistically significant relations between EF and TAA/TNA, IVST and TAA/TNA, fasting blood sugar and TAA/TNA, creatinine and TAA/TNA, HDL and TAA/TNA, and hemoglobin (g/dL) and TAA/TNA were detected. Additionally, statistically significant relations between EF and the mean AgNOR number, IVST and the mean AgNOR number, fasting blood sugar and the mean AgNOR number, creatinine and the mean AgNOR number, LDL (mg/dL) and the mean AgNOR number, HDL (mg/dL) and the mean AgNOR number, and hemoglobin (g/dL) and the mean AgNOR number were detected. Considering the relationship between interventricular septum thickness and hypertension, the statistically significant relationship between the TAA/TNA ratio, the mean AgNOR number, and cardiovascular risk factors such as hypertension, dyslipidemia, high blood sugar, creatinine, and anemia suggests that AgNOR proteins may have some important roles that we have not yet identified in the pathophysiology of atherosclerotic cardiovascular disease.

In addition, we found that both the mean AgNOR number and the TAA/TNA ratio increased as the EF decreased. Based on this, it can be said that AgNOR parameters increase as the severity of myocardial infarction increases or the damage to myocardial tissue increases. According to the current study, the mean AgNOR number allowed 86.7% sensitivity and 86.2% specificity and the TAA/TNA ratio allowed 98.7% sensitivity and 98.5% specificity for the discrimination of the control group from the STEMI group (Table 4).

According to the literature, high WBC levels are considered an independent predictor of acute myocardial infarction because of their association with more complications, more extensive myocardial necrosis, and worse outcomes [18,19,20,21]. In our study, in line with the literature, WBC count, neutrophil count, neutrophil/lymphocyte ratio, and monocyte count were significantly higher in STEMI patients compared to the control group.

In addition, from the data of our study, we found a statistically significant relationship between both AgNOR parameters (TAA/TNA and the mean AgNOR number) and WBCs, neutrophils, the neutrophil/lymphocyte ratio, and monocytes. The correlation between AgNOR proteins and parameters such as WBC count, neutrophil count, neutrophil/lymphocyte ratio, and monocyte count, which are predictors of cardiovascular mortality and morbidity in patients with STEMI, may open the door to a new marker in STEMI. As is known in the diagnosis of STEMI, the fastest diagnostic tool is undoubtedly ECG. In addition, other parameters such as troponin, CRP, and WBC can provide useful information about the prognosis of the disease. Both AgNOR protein amounts increase depending on the hypoxia that occurs in STEMI. Considering that AgNOR proteins are a secondary response to inflammation, they are likely to provide some prognostic information. In addition, if this work is supported by larger studies in the future, it may benefit diagnosis and prognosis in the non-ST-elevation myocardial infarction (NSTEMI) patient group.

The limitations of present study are the relatively small study population and the single-center nature of the study.

## 5. Conclusions

In conclusion, we suggest that AgNOR parameters could be a reliable marker in patients with STEMI. Prospective studies with a larger cohort are required to establish its prognostic role in this population.

## Figures and Tables

**Table 1:** Demographic, laboratory, and echocardiographic findings of both groups.

	STEMI (*n* = 75) Mean ± SD (Min–Max) (Median)	Control (*n* = 65) Mean ± SD (Min–Max) (Median)	χ^2^	*p*
Sex (M/F) (%)	60 (80%)/15 (20%)	52 (80%)/13 (20%)	0.000	1.000
Diabetes mellitus (Yes/No) (%)	36 (48%)/39 (52%)	21 (32.3%)/44 (67.7%)	3.552	0.059
Hypertension (Yes/No) (%)	40 (53.3%)/35 (46.7%)	37 (56.9%)/28 (43.1%)	0.81	0.670
Hyperlipidemia (Yes/No) (%)	38 (50.7%)/37 (49.3%)	33 (50.8%)/32 (49.2%)	0.000	0.990
FHCD (Yes/No) (%)	38 (50.7%)/37 (49.3%)	27 (41.5%)/38 (58.5%)	1.168	0.280
Smoking (Yes/No) (%)	37 (49.3%)/38 (50.7%)	31 (47.7%)/34 (52.3%)	0.038	0.846
			**Z**	***p***
Age (years)	58.96 ± 10.37 (34–75) (59)	56.29 ± 10.14 (36–75) (57)	−1.519	0.129
BMI at diagnosis (kg/m^2^)	27.86 ± 1.74 (21.78–32.08) (27.78)	27.62 ± 3.16 (20.96–34.72) (27.31)	−1.216	0.224
Systolic blood pressure (mmHg)	135.33 ± 9.74 (100–160) (140)	134.46 ± 12.09 (110–160) (140)	−0.362	0.717
Diastolic blood pressure (mmHg)	86.47 ± 6.03 (60–100) (90)	85.62 ± 7.04 (70–100) (85)	−1.065	0.224
Fasting blood sugar (mg/dL)	117.81 ± 30.49 (76–175) (104)	106.58 ± 27.44 (70–170) (95)	−1.996	0.046
Creatinine (mg/dL)	0.94 ± 0.21 (0.48–1.40) (0.91)	0.85 ± 0.15 (0.5–1.2) (0.86)	−2.272	0.023
LDL (mg/dL)	112.55 ± 38.48 (28–211) (109)	118.32 ± 36.27 (49–212) (118)	−0.986	0.324
HDL (mg/dL)	38.77 ± 9.13 (21–59) (38)	44.63 ± 10.25 (23–70) (45)	−3.474	0.001
Triglyceride (mg/dL)	186.81 ± 148.73 (48–900) (144)	159.54 ± 94.58 (47–594) (135)	−0.345	0.730
Total cholesterol (mg/dL)	186.25 ± 50.14 (93–359) (181)	196.55 ± 44.17 (120–320) (193)	−1.617	0.106
Hemoglobin (g/dL)	13.54 ± 1.54 (10–16.9)	14.20 ± 1.53 (10–16.8)	−2.663	0.008
WBC (µL/mL)	11,410.67 ± 2942.68 (5700–19,200) (12,000)	7013.85 ± 1652.16 (4100–10,900) (6800)	−8.103	<0.001
Platelet (×10^3^)	249.39 ± 57.65 (137–432) (255)	261.78 ± 61.98 (142–467) (253)	−0.913	0.361
Lymphocyte (×10^3^)	2.35 ± 1.48 (0.37–8) (2)	2.18 ± 0.61 (0.79–4.15) (2.18)	−0.493	0.622
Monocyte (×10^3^)	0.80 ± 0.44 (0.1–2.6) (0.75)	0.53 ± 0.16 (0.24–1.02) (0.53)	−4.607	<0.001
Neutrophil (×10^3^)	8.19 ± 2.77 (3.41–15.50) (8.2)	4.06 ± 1.17 (1.29–6.97) (4.1)	−8.586	<0.001
Neutrophil/lymphocyte	5.33 ± 5.26 (0.84–31.57) (3.68)	1.99 ± 0.88 (0.57–6.97) (1.89)	−6.211	<0.001
Mean AgNOR number	2.56 ± 0.8 (1.23–5) (2.5)	1.32 ± 0.49 (1–3) (1)	−8.716	<0.001
TAA/TNA	0.11 ± 0.03 (0.04–0.18) (0.11)	0.03 ± 0.01 (0.02–0.06) (0.03)	−10.104	<0.001
**Echocardiographic Findings**				
	**STEMI Mean ± SD (Min–Max)**	**Control Mean ± SD (Min–Max)**	**Z**	***p***
IVST (cm)	1.16 ± 0.12 (0.9–1.5) (1.2)	1.08 ± 0.13 (0.9–1.5) (1)	−4.012	<0.001
EF (*n* %)	47.69 ± 7.89 (20–60) (50)	63.62 ± 2.91 (50–65) (65)	−10.051	<0.001
	STEMI *n* (%)	Control *n* (%)	χ;^2^	*p*
MR (Yes/No) (%)	52 (69.3%)/23 (30.7%)	21 (32.3%)/44 (67.7%)	19.130	<0.001
AR (Yes/No) (%)	12 (17.3%)/62 (82.7%)	7 (10.58%)/58 (89.2%)	1.225	0.268
PR (Yes/No) (%)	12 (16%)/63 (84%)	5 (7.7%)/60 (92.3%)	2.253	0.133
TR (Yes/No) (%)	45 (60%)/30 (40%)	24 (36.9%)/41 (63.1%)	7.419	0.006

**BMI**: Body mass index; **FHCD**: family history of cardiovascular disease; **HDL**: high-density lipoprotein; **LDL**: low-density lipoprotein; **WBC**: white blood cells; **MR**: mitral regurgitation; **AR**: aortic regurgitation; **TR**: tricuspid regurgitation; **PR**: pulmonary regurgitation; **IVST**: interventricular septum thickness; **Min**–**Max**: minimum–maximum; **SD**: standard deviation; **TAA**: total AgNOR area; **TNA**: total nuclear area; **AgNOR**: argyrophilic nucleolar-organizing region; **EF**: ejection fraction. The cardiovascular risk factors are shown in Table 2.

**Table 2:** Model summary and parameter estimates for TAA/TNA and IVST, fasting blood sugar, creatinine, HDL, hemoglobin, WBC, monocyte, neutrophil, and neutrophil/lymphocyte ratio of both groups.

		Model Summary					Parameter Estimates			
Variable	Equation	R^2^	F	df1	df2	sig	Constant	b1	b2	b3
IVST and TAA/TNA	Linear	0.102	15.615	1	138	<0.001	1.049	0.943		
	Log	0.090	13.574	1	138	<0001	1.289	0.061		
	Cubic	0.113	5.759	3	136	0.001	1.178	−4.961	6.959	−233.473
Fasting blood sugar and TAA/TNA	Linear	0.039	5.564	1	138	0.020	102.487	132.121		
	Log	0.038	5.511	1	138	0.020	137.498	9.020		
	Cubic	0.040	1.892	3	136	0.134	93.230	536.768	−4423.553	13,886.456
Creatinine and TAA/TNA	Linear	0.043	6.237	1	138	0.014	0.828	0.894		
	Log	0.039	5.627	1	138	0.019	1.057	0.058		
	Cubic	0.070	3.387	3	136	0.020	1.071	−10.944	148.978	−542.843
HDL (mg/dL) and TAA/TNA	Linear	0.061	9.020	1	138	0.003	45.829	−56.646		
	Log	0.069	10.169	1	138	0.002	30.151	−4.109		
	Cubic	0.088	4.379	3	136	0.006	42.925	145.671	−3452.466	15,606.463
Hemoglobin (g/dL) and TAA/TNA	Linear	0.036	5.204	1	138	0.024	14.368	−6.789		
	Log	0.045	6.427	1	138	0.012	12.426	−0.515		
	Cubic	0.052	2.462	3	136	0.065	14.738	−15.514	−26.853	554.055
WBC (µL/mL) and TAA/TNA	Linear	0.358	76.924	1	138	<0.001	5965.689	44,466.344		
	Log	0.373	81.993	1	138	<0.001	17,959.856	3112.044		
	Cubic	0.406	31.038	3	136	<0.001	7449.484	−53,605.833	1,618,705.263	−7,182,084.69
Monocyte (×10^3^) and TAA/TNA	Linear	0.096	14.727	1	138	<0.001	0.478	2.560		
	Log	0.099	15.123	1	138	<0.001	1.164	0.178		
	Cubic	0.111	5.670	3	136	0.001	0.652	−7.112	139.691	−569.051
Neutrophil (×10^3^) and TAA/TNA	Linear	0.344	72.240	1	138	<0.001	3.210	40.017		
	Log	0.372	81.584	1	138	<0.001	14.151	2.854		
	Cubic	0.425	33.512	3	136	<0.001	4.238	−43.604	1550.651	−7314.028
Neutrophil/ lymphocyte and TAA/TNA	Linear	0.130	20.698	1	138	<0.001	1.128	34.698		
	Log	0.139	22.363	1	138	<0.001	10.577	2.461		
	Cubic	0.172	9.410	3	136	<0.001	3.799	−122.341	2379.721	−10,018.401
EF and TAA/TNA	Linear	0.509	142.807	1	138	0.000	67.518	−162.426		
	Log	0.549	167.926	1	138	0.000	23.139	−11.573		
	Cubic	0.572	60.493	3	136	0.000	75.085	−405.136	1204.423	1787.206

**IVST**: Interventricular septum thickness; **EF**: ejection fraction; **WBC**: white blood cell; **HDL**: high-density lipoprotein; **TAA**: total AgNOR area; **TNA**: total nuclear area; **AgNOR**: argyrophilic nucleolar-organizing region.

**Table 3:** Model summary and parameter estimates for the mean AgNOR number and IVST, fasting blood sugar, creatinine, LDL, HDL, hemoglobin, WBCs, monocytes, neutrophils, and neutrophil/lymphocyte ratio of both groups.

		Model Summary					Parameter Estimates			
Variable	Equation	R^2^	F	df1	df2	sig	Constant	b1	b2	b3
IVST and mean AgNOR	Linear	0.068	10.014	1	138	0.002	1.048	0.037		
	Log	0.061	8.983	1	138	0.003	1.081	0.070		
	Cubic	0.069	3.360	3	136	0.021	1.093	−0.021	0.021	−0.002
Fasting blood sugar and mean AgNOR	Linear	0.045	6.565	1	138	0.011	98.958	6.875		
	Log	0.040	5.719	1	138	0.018	105.125	12.857		
	Cubic	0.048	2.290	3	136	0.081	106.272	−0.889	1.763	−0.024
Creatinine and mean AgNOR	Linear	0.022	3.113	1	138	0.080	0.835	0.031		
	Log	0.014	2.022	1	138	0.157	0.867	0.050		
	Cubic	0.062	2.997	3	136	0.033	0.718	0.269	−0.132	0.021
LDL (mg/dL) and mean AgNOR	Linear	0.056	8.205	1	138	0.005	134.453	−9.686		
	Log	0.051	7.408	1	138	0.007	125.951	−18.438		
	Cubic	0.068	3.283	3	136	0.023	162.666	−56.427	22.083	−3.059
HDL (mg/dL) and mean AgNOR	Linear	0.072	10.783	1	138	0.001	47.366	−2.960		
	Log	0.086	12.950	1	138	<0.001	45.233	−6.433		
	Cubic	0.094	4.702	3	136	0.004	58.798	−17.121	4.00	−0.396
Hemoglobin (g/dL) and mean AgNOR	Linear	0.078	11.638	1	138	0.001	14.796	−0.477		
	Log	0.083	12.499	1	138	0.001	14.422	−0.986		
	Cubic	0.103	5.201	3	136	0.002	17.819	−4.878	1.14	−0.221
WBC (µL/mL) and mean AgNOR	Linear	0.170	28.298	1	138	<0.001	6444.631	1473.871		
	Log	0.205	35.527	1	138	<0.001	7491.555	3229.917		
	Cubic	0.222	12.970	3	136	<0.001	1688.337	7049.536	−1626.10	108.735
Monocyte (×10^3^) and mean AgNOR	Linear	0.033	4.706	1	138	0.032	0.531	0.072		
	Log	0.046	6.694	1	138	0.011	0.575	0.170		
	Cubic	0.079	3.915	3	136	0.010	0.452	0.035	0.091	−0.021
Neutrophil (×10^3^) and mean AgNOR	Linear	0.223	39.523	1	138	<0.001	3.201	1.548		
	Log	0.250	46.118	1	138	<0.001	4.366	3.281		
	Cubic	0.256	15.635	3	136	<0.001	0.078	5.073	−0.944	0.047
Neutrophil/lymphocyte ratio and mean AgNOR	Linear	0.099	15.237	1	138	<0.001	0.894	1.456		
	Log	0.104	16.073	1	138	<0.001	2.051	2.980		
	Cubic	0.108	5.515	3	136	0.001	1.545	−0.244	1.076	−0.181
EF and mean AgNOR	Linear	0.260	48.550	1	138	<0.001	66.169	−5.585		
	Log	0.315	63.584	1	138	<0.001	62.228	−1.285		
	Cubic	0.351	24.483	3	136	<0.001	92.315	−38.908	11.372	−1.070

**IVST**: Interventricular septum thickness; **WBC**: white blood cell; **HDL**: high-density lipoprotein; **LDL**: low-density lipoprotein; **AgNOR**: argyrophilic nucleolar-organizing region; **EF**: ejection fraction.

**Table 4:** Bayesian statistics based on ROC-derived cut-off values.

	Groups	AUC (95%)	Cut-Off	*p*	Sensitivity (%)	Specificity (%)
**Mean AgNOR number**	STEMI: 75 and Control: 65	0.923 (0.879–0.967)	1.523	0.000	86.7	86.2
**TAA/TNA**		0.996 (0.988–1)	0.054	0.000	98.7	98.5

**AUC**: Area under the ROC curve; **TAA**: total AgNOR area.

## Data Availability

The data presented in this study are available on request from the corresponding author. The data are not publicly available since the people participating in the study have not allowed their data to be shared with third parties.
